# Semi-IPN Films and Electrospun Nanofibers Based on Chitosan/PVA as an Antibacterial Wound Dressing

**DOI:** 10.22037/ijpr.2019.1100712

**Published:** 2019

**Authors:** Keshvad Hedayatyanfard, Shadab Bagheri-Khoulenjani, Ali Hashemi, Seyed Ali Ziai

**Affiliations:** a *Students Research Committee, Department of Pharmacology, School of Medicine, Shahid Beheshti University of Medical Sciences, Tehran, Iran. *; b *Department of Polymer Engineering, Faculty of Biomedical Engineering, Amirkabir University of Technology, Tehran, Iran.*; c *Department of Microbiology, School of Medicine, Shahid Beheshti University of Medical Sciences, Tehran, Iran.*; d *Department of Pharmacology, School of Medicine, Shahid Beheshti University of Medical Sciences, Tehran, Iran.*

**Keywords:** PVA, Chitosan, Doxycycline, Film, Nanofiber, Antimicrobial, Genipin

## Abstract

The antimicrobial activity of a wound dressing is a key factor for preventing and treating wound infection. The current study evaluated the physiochemical properties and antimicrobial activities of semi-IPNs (interpenetrating polymer networks) based on chitosan/polyvinyl alcohol (PVA) films and nanofibers as candidates for wound dressings and investigated the effects of morphologies (nanofibrous mats and films), crosslinking conditions of chitosan chains (uncrosslinked and crosslinked with genipin), and the presence of antibacterial drug (doxycycline) on their physicochemical and antibacterial properties. The morphology, chemical structure, fluid uptake, water vapor transmission rate, antimicrobial activity, and doxycycline release profile were assayed using SEM, FTIR spectroscopy, swelling test, permeation test, agar diffusion antibiogram, and dissolution test, respectively. The results demonstrated that crosslinking chitosan with genipin reduced the diameter of nanofibers, fluid uptake, and drug release from both nanofiber mats and film samples. According to the results, wound dressings with film morphology have better antimicrobial activity than those with nanofiber. The chitosan/PVA/Doxycycline 1% film has the potential for use as an antimicrobial wound dressing.

## Introduction

An ideal wound dressing contains features like antimicrobial activity, sustained drug release into the wound, gaseous exchange capability, non-toxicity, biocompatibility, absorption of exudate, and ease of removal (1-3). Bacterial colonization on the wound environment leads to prolonged inflammation and delay in wound healing (4, 5). Using an appropriate antibiotic to prevent the accumulation and growth of bacteria is essential. Antimicrobial compounds used in wound dressing are silver, honey, iodine, and antibiotics such as metronidazole, neomycin, gentamycin, and mupirocin (1, 6 and 7). Chitosan is a non-toxic, biodegradable, biocompatible, and non-antigenic polymer derived from D-glucosamine and N-acetyl-D-glucosamine (8, 9). It has antibacterial properties (10). The antimicrobial activity of chitosan depends on molecular weight, concentration, positive charge density, chelating capacity, pH, temperature, hydrophilic /hydrophobic properties, and physical form (11). 

Doxycycline is a semisynthetic tetracycline. The antibacterial mechanism of doxycycline is the ability to inhibit aminoacyl-tRNA attachment to ribosome (12). In addition, tetracycline antibiotics can suppress cell proliferation and inflammation, modulate the immune system and angiogenesis (13-16). Its antibiotic and non-antibiotic properties and ability to promote wound healing make doxycycline a suitable drug for wound dressing. Doxycycline has been added to various forms of chitosan such as sponge, microsphere, and hydrogel to improve their antibacterial activity and wound healing effects (17-19). Crosslinking chitosan with linkers such as genipin can control drug release (20). Genipin is a strong cross linker, and it is biocompatible with many polymers such as chitosan and proteins (21). There are various techniques for the fabrication of wound dressing. Among them the most popular are film preparation and electrospinning. Electrospinning is a technique used in the production of nanofibers from polymer solutions using electrostatic forces (22). A nanofiber wound dressing has unique properties in comparison with other wound dressings. Examples of such properties are their high surface-to-volume ratio and high porosity which lead to increased cell-matrix interaction (23). Film wound dressings are flexible, transparent, permeable to water vapor and oxygen, and impermeable to water and bacteria (24). The casting/solvent evaporation method has been used to fabricate a film wound dressing (25, 26). In the current study, electrospinning and casting methods were used for the preparation of films and a nanofibrous mat of chitosan/PVA/doxycycline, and their physiochemical and antimicrobial properties were compared.

## Experimental

Low molecular weight chitosan with deacetylation degrees (DD) of 75–85% and poly (vinyl alcohol) (PVA) (M_w_ 89000-98000, 99% hydrolyzed) were purchased from the Sigma Aldrich Co., USA. Acetic acid was purchased from Merck Co., Germany. Doxycycline hyclate was supplied by Sigma Co., USA. Genipin was purchased from Challenge Bioproducts Co., LTD, Taiwan. 

*Film preparation*

Four different film (f) formulations: f-chitosan (C)/poly(vinyl alcohol) (P), f-C/P/doxycycline (D) 1%, f-C/P/ D_1%_/genipin (G) 0.05%, and f-C/P/D_1%_/G_0.1% _were fabricated using the casting method (Table 1). Chitosan powder 3% (w/v) was dispersed in acetic acid 1% for 3 h at 25 °C while being stirred at 250 rpm. PVA powder 5% (w/v) was dispersed in distilled water (120 °C) with intense stirring for 3 h. After degassing, C/P solutions 20/80 (v/v) were mixed for 18 h at 25 °C. To prepare semi-IPN films, doxycycline 1% (w/w) was dissolved in distilled water, and genipin 0.05% or 0.1% (w/w) was dissolved in 0.5 mL ethanol 90 ° for 1 h and added to the chitosan/PVA solution. The final solution was poured into a circular cast and dried at room temperature for 3 days.

*Nanofiber preparation*

Chitosan powder 3% (w/v) was stirred at 250 rpm in acetic acid 70% (v/v) for 3 h at 25 °C. PVA powder 10% (w/v) was dispersed in distilled water at 120 °C with intense stirring for 3 h. After degassing the solutions, chitosan and PVA with a ratio of 20/80 (v/v) was mixed overnight for 18 h at 25 °C. In order to fabricate semi-IPN nanofibers, doxycycline 1% (w/w) was dissolved in distilled water, genipin 0.05% or 0.1% (w/w) was dissolved in 0.5 mL ethanol at 90 °, and ethanol was added to the chitosan/PVA solutions. Four different formulation solutions were prepared: nanofiber (n)-C/P, n-C/P/D_1%_, n-C/P/D_1%_/G_0.05%_, and n-C/P/D_1%_/G_0.1%_ (Table 1). The polymer solutions were inserted into 5 mL glass syringe with a stainless steel 19-gauge needle, of which the distance between tip and aluminum collector was 15 cm. High voltage power (20 kV) was applied, and the feed rate for injection of the solution was 0.3 mL/h. All procedures were conducted at room temperature, and the solutions were dried for 3 days at 25 °C.

*FT-IR and ATR analysis*

The chemical structure of the nanofiber samples was recorded with FT-IR (Thermo*-*Nicolet Nexus 670, USA) at a wavenumber range of 400-4000 cm^-1^, and attenuated total reflection (ATR) was performed for the film samples (Smart ARK, USA) with a wavenumber range of 650-4000 cm^-1^.

In ambient conditions and laboratory temperature, a specific proportion of the samples and potassium bromide powder were mixed under pressure to prepare pellets. The prepared pellets were inserted into the FT-IR device for analysis. For FTIR-ATR, the samples were placed on the surface of ZnSe crystal and inside the chamber for analysis.

*Scanning electron microscopy (SEM)*

The surface morphology of film samples and the diameter of the nanofiber mats were determined using scanning electron microscopy (SEM) (AIS2100, South Korea) at voltage of 20 kV. All samples were sputter coated with gold for 30 min. The nanofiber diameters were measured with an image processing software.

*Water uptake measurements*

To measure water uptake of the wound dressings, circular samples with a diameter of 15 mm were cut, weighted (W_1_), and placed into the dishes with 50 mL PBS (pH 7.4) at room temperature. After 4 h, the samples were taken out and excess PBS was removed with filter paper and weighted (W_2_). For each sample, the experiment was repeated 3 times and the mean ± SD was reported. The water uptake was calculated using the following formula:

 Water uptake percentage = ((W_2 _- W_1_)/W_1_) × 100                     (1) 

*Water vapor transmission rate (WVTR) measurement*

Circular samples of films and nanofibers were placed on the top of glass tubes containing PBS (pH 7.4) and fixed. Afterwards the glass tubes were weighted (W_0_) and inserted into a glass jar containing 1 kg fresh silica gel and placed in the incubator at 37 °C. At appropriate time intervals, the tubes were weighted (W_1_) and the WVTR was calculated from the line slope of weight changes where, according to Equation 2, area = exposure area of tubes and slope = weight changes relative to time (27).

WVTR = [g/m^2 ^× day] [slope × 24/area]                      (2)

*In-vitro release study*

The release of doxycycline from nanofibers and films was simulated by dissolving a similar exposed disc (6 mm) in a suitable volume (20 mL) of PBS (pH 7.4) in glass vessels. The glass vessels were kept in the shaker set at 64 rpm at 37 °C for 48 h. The specific volumes of the solution were collected from the glass vessels at specific time intervals and assayed by UV spectrophotometry (Analytik Jena, Germany) at 276 nm. An equal volume of PBS was added back into glass vessels to replace the removed volume.

*Antimicrobial susceptibility study*

To evaluate the antimicrobial activity of different forms of nanofiber mats and films, circular samples with a diameter of 6 mm were cut and sterilized by UV light for 30 min. The organisms used to examine the antimicrobial activity of the wound dressings were standard bacterial strains of *P. aeruginosa* (*Pseudomonas aeruginosa*) PAO1, *S*.* aureus* (*Staphylococcus aureus*) ATCC 25923, and *A*.* baumannii* (*Acinetobacter baumannii*) ATCC19606. The antibacterial activity was determined according to CLSI (Clinical Laboratory Standards Institute) 2016 guidelines. Bacterial colonies were suspended in Mueller-Hinton broth (Merck, Germany) to reach a turbidity equal to the 0.5 McFarland standards. The bacterial suspension was inoculated on the surface of Mueller-Hinton agar (Merck, Germany) plates using a sterile swap. All sample discs of nanofibers and film were placed on the surface of plates. After incubation at 37 °C for 24 h, the diameter of the inhibition zone for each sample was measured in millimeters (mm) using a ruler.

*Statistical analysis*

Data was presented as mean ± SD, and all analyses were performed using GraphPad Prism software. A *p*-value of ˂0.05 was considered significant.

## Results and Discussion

*Morphological studies*

**Figure 1:**
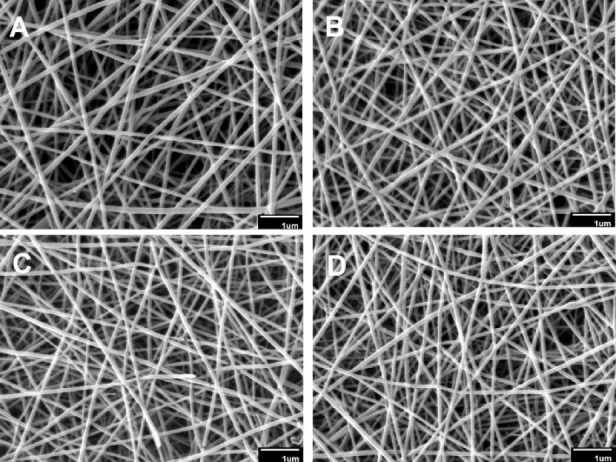
The SEM diagrams of nanofiber mats: (A) n-C/P (nanofiber-Chitosan/PVA), (B) n-C/P/D1% (nanofiber-Chitosan/PVA/ Doxycycline 1%), (C) n-C/P/D1%/G0.05% (nanofiber-Chitosan/PVA/Doxycycline 1%/Genipin 0.05%) and (D) n-C/P/D1%/G0.1% (nanofiber- Chitosan/PVA/Doxycycline 1%/Genipin 0.1%).

**Figure 2:**
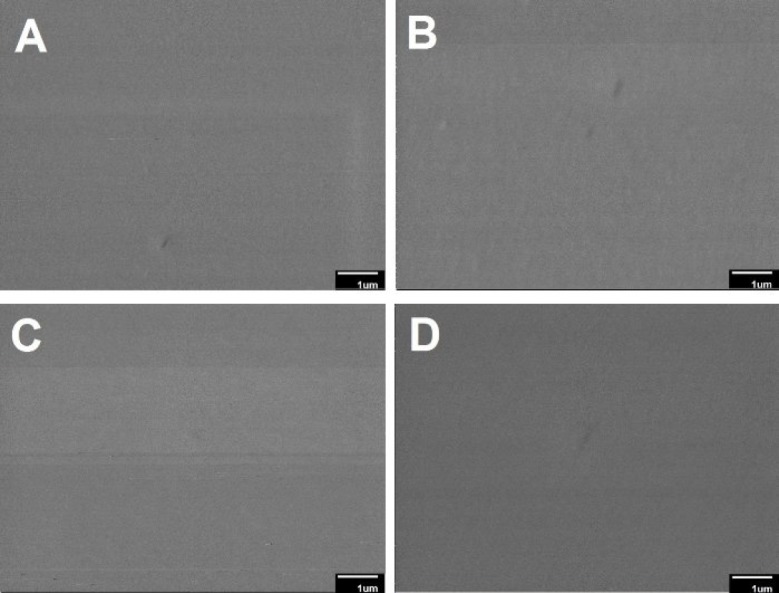
The SEM diagrams of film samples: (A) f-C/P (film-Chitosan/PVA), (B) f-C/P/D1% (film- Chitosan/PVA/Doxycycline 1%),(C) f-C/P/D1%/G0.05% (film-Chitosan/PVA/Doxycycline 1%/Genipin 0.05%) and (D) f-C/P/D1%/G0.1% (film-Chitosan/PVA/Doxycycline 1%/Genipin 0.1%).

The morphology and mean diameter of nanofibers are shown in Table 2 and Figure 1. The electrospinning device variables (voltage, the distance between tip and aluminum collector, feed rate, and aluminum collector speed) were adjusted to obtain an optimal nanofibrous mat without beads. The results indicated that the diameter of the crosslinked nanofibers was non-significantly lesser than that of the uncrosslinked nanofibers (*p *> 0.05). These results were in agreement with those of Norowski *et al.*, who demonstrated that genipin decreased the diameter of chitosan nanofibers (28). In the current study, it was also found that the addition of doxycycline decreased non-significantly the diameter of nanofibers (n-C/P/D_1%_ = 100 nm *vs.* n-C/P = 104 nm [*p *> 0.05]). Doxycycline hydrochloride may reduce the viscosity and increase the conductivity of the electrospinning solution, causing a reduction in the diameter of the nanofibers. The small size of the nanoscale fiber increases the cell-matrix interaction (23). SEM images of the wound dressing films, prepared using the film casting method showed that the changes in the percentage of genipin and the presence of doxycycline in the structure of films had no effect on surface morphology or roughness (Figure 2).

*Chemical structure of films and nanofibers*

**Figure 3:**
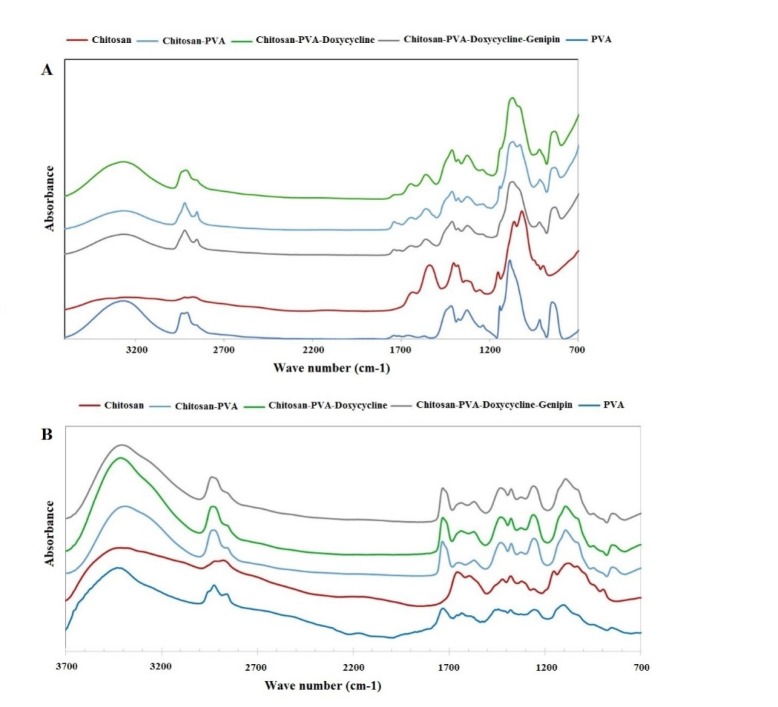
(A) The ATR-FTIR of (B) films and FTIR of nanofibers

ATR-FTIR and FTIR were used to evaluate the chemical structure of the wound dressings prepared by film casting and electrospinning methods, respectively (29-31). Figure 3A shows the ATR-FTIR spectra of film formulations, raw chitosan, and raw PVA. Chitosan peaks were observed at 897 and 1151 cm^-1^ (pyranose structure), 1066 cm^-1^ (C-O stretching), 1321 cm^-1^ (vibration of CH), 1540 cm^-1^ (NH bending of NH2), 1625 cm^-1^ (C=O of amide bond), 2916 cm^-1^ (CH vibration), and 3415 cm^-1^ (-OH hydrogen bonds and –NH stretching). PVA peaks were shown at 3265 cm^-1^ (OH hydrogen bonds), 2936 cm^-1^ (CH vibrations), 1739 (C=O of remaining acetate groups), and 1084 and 1416 cm^-1^ (C-O groups). Figure 3A shows all the peaks reported above were observed in the spectrum of the PVA/chitosan samples. However, the peak at 3416 cm^-1^ in the chitosan spectrum of NH and OH was shifted to 3266 cm^-1^. Also, the peak observed at1654 cm^-1^ in the chitosan spectrum was shifted to 1647 cm^-1^ after PVA was added to the formulation. These shifts show the formation of strong hydrogen bonds between PVA and the chitosan chains. The addition of doxycycline to the chitosan/PVA formulation shifted the peak observed at 1647 cm^-1^ to 1643 cm^-1^. This may be explained by the hydrogen binding of the C=O group of doxycycline with PVA and the chitosan chains. 

The addition of genipin did not change the position of the peaks significantly. This may be explained by the overlaps of the new amide formed via the interaction between the NH2 groups of chitosan with genipin and those already existing in the chitosan structure. Genipin molecules interacted only with the NH2 groups and not with the OH groups of PVA. Figure 3B shows the FTIR spectra of electrospun nanofibers. The same trend was observed in FTIR spectra of the nanofibrous samples.

**Table 1:** Nanofibers and films wound dressing concentration. n-C/P (nanofiber-Chitosan/PVA), n-C/P/D1% (nanofiber-Chitosan/PVA/ Doxycycline 1%), n-C/P/D1%/G0.05% (nanofiber-Chitosan/PVA/Doxycycline 1%/Genipin 0.05%), n-C/P/D1%/G0.1% (nanofiber-Chitosan/ PVA/Doxycycline 1%/Genipin 0.1%), f-C/P (film-Chitosan/PVA), f-C/P/D1% (film- Chitosan/PVA/Doxycycline 1%), f-C/P/D1%/G0.05% (film-Chitosan/PVA/Doxycycline 1%/Genipin 0.05%), f-C/P/D1%/G0.1% (film-Chitosan/PVA/Doxycycline 1%/Genipin 0.1%).

**Wound dressing**	**Chitosan (C)**	**PVA (P)**	**Doxycycline (D)**	**Genipin (G)**
Film casting				
f-C/P	3%	5%	0%	0%
f-C/P/D1%	3%	5%	1%	0%
f-C/P/D1%/G0.05%	3%	5%	1%	0.05%
f-C/P/D1%/G0.1%	3%	5%	1%	0.1%
Electrospinning				
n-C/P	3%	5%	0%	0%
n-C/P/D1%	3%	5%	1%	0%
n-C/P/D1%/G0.05%	3%	5%	1%	0.05%
n-C/P/D1%/G0.1%	3%	5%	1%	0.1%

**Table 2:** The diameter (nm) of nanofiber mats. n-C/P (nanofiber-Chitosan/PVA), n-C/P/D1% (nanofiber-Chitosan/ PVA/Doxycycline 1%), n-C/P/D1%/G0.05% (nanofiber-Chitosan/ PVA/Doxycycline 1%/Genipin 0.05%) and n-C/P/D1%/G0.1% (nanofiber-Chitosan/PVA/Doxycycline 1%/Genipin 0.1%).

**Nanofiber mats**	**Mean ± SD**
n-C/P	104 ± 24.99
n-C/P/D1%	100 ± 15.68
n-C/P/D1%/G0.05%	94 ± 16.45
n-C/P/D1%/G0.1%	94 ± 17.63

**Figure 4:**
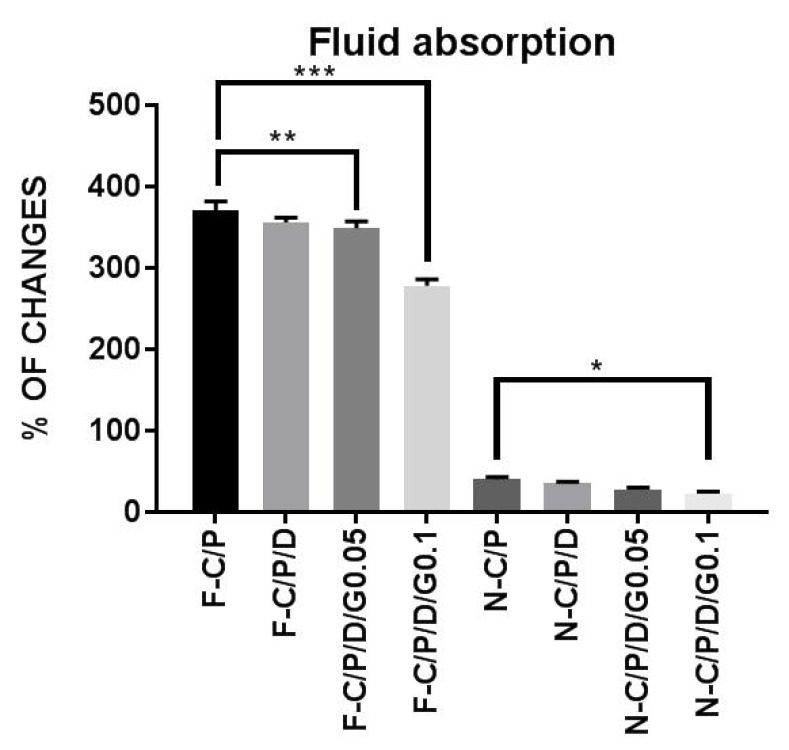
Water uptake of the nanofiber mats and films**. **n-C/P (nanofiber-Chitosan/PVA), n-C/P/D1% (nanofiber-Chitosan/PVA/ Doxycycline 1%), n-C/P/D1%/G0.05% (nanofiber-Chitosan/PVA/Doxycycline 1%/Genipin 0.05%), n-C/P/D1%/G0.1% (nanofiber-Chitosan/ PVA/Doxycycline 1%/Genipin 0.1%), f-C/P (film-Chitosan/PVA), f-C/P/D1% (film- Chitosan/PVA/Doxycycline 1%), f-C/P/D1%/G0.05% (film-Chitosan/PVA/Doxycycline 1%/Genipin 0.05%), f-C/P/D1%/G0.1% (film-Chitosan/PVA/Doxycycline 1%/Genipin 0.1%)

**Figure 5:**
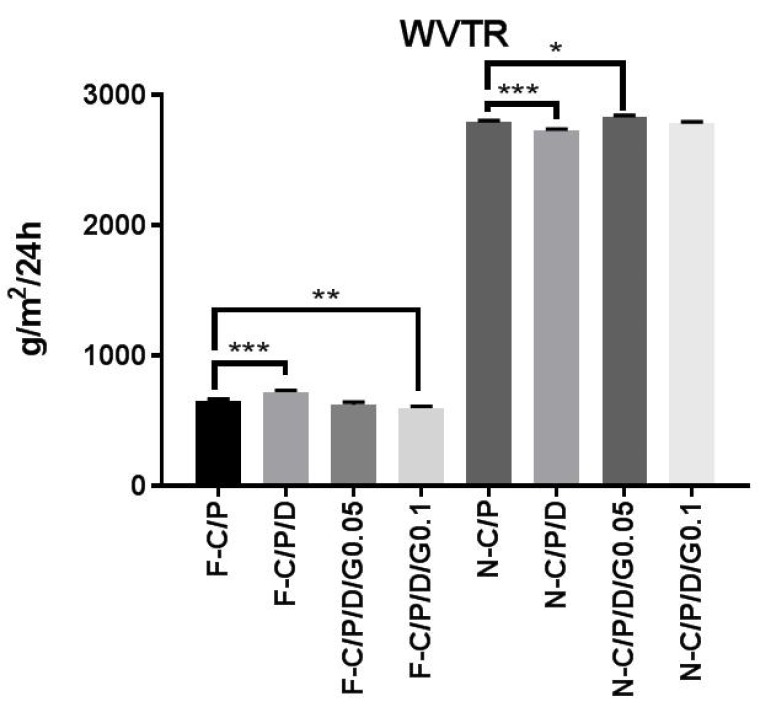
Water vapor transmission rate (WVTR) of the nanofiber mats and films**. **n-C/P (nanofiber-Chitosan/PVA), n-C/P/ D1% (nanofiber-Chitosan/PVA/Doxycycline 1%), n-C/P/D1%/G0.05% (nanofiber-Chitosan/PVA/Doxycycline 1%/Genipin 0.05%), n-C/P/ D1%/G0.1% (nanofiber-Chitosan/PVA/Doxycycline 1%/Genipin 0.1%), f-C/P (film-Chitosan/PVA), f-C/P/D1% (film- Chitosan/PVA/ Doxycycline 1%), f-C/P/D1%/G0.05% (film-Chitosan/PVA/Doxycycline 1%/Genipin 0.05%), f-C/P/D1%/G0.1% (film-Chitosan/PVA/ Doxycycline 1%/Genipin 0.1%).

**Figure 6:**
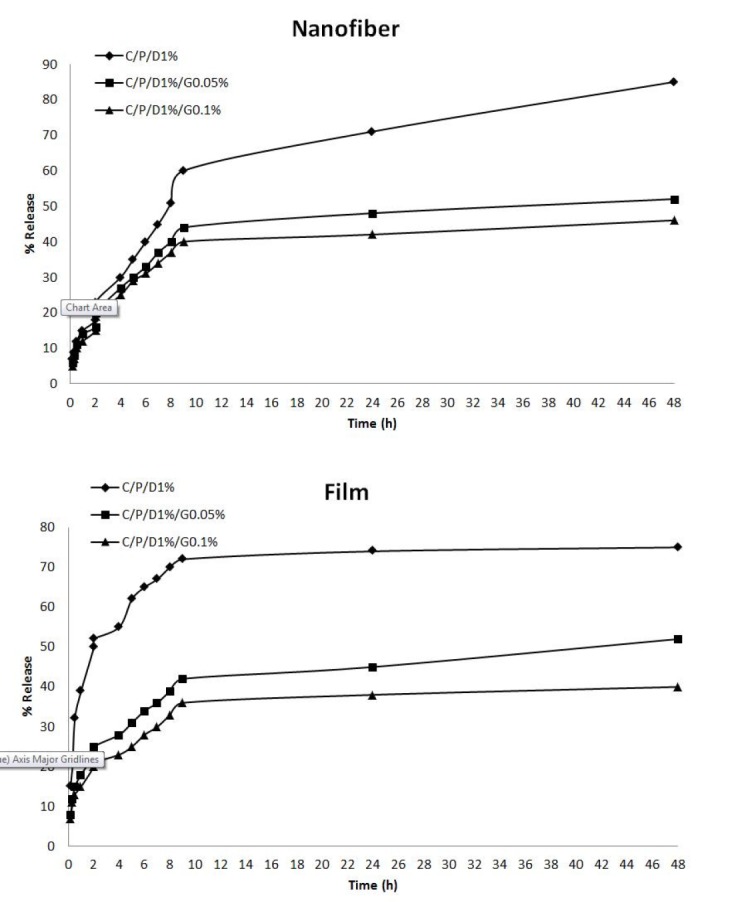
Release profile of doxycycline from wound dressing. n-C/P/D1% (nanofiber-Chitosan/PVA/Doxycycline 1%), n-C/P/D1%/G0.05% (nanofiber-Chitosan/PVA/Doxycycline 1% /Genipin 0.05%) and n**-**C/P/D1%/G0.1% (nanofiber-Chitosan/PVA/Doxycycline 1% /Genipin 0.1%).

**Figure 7:**
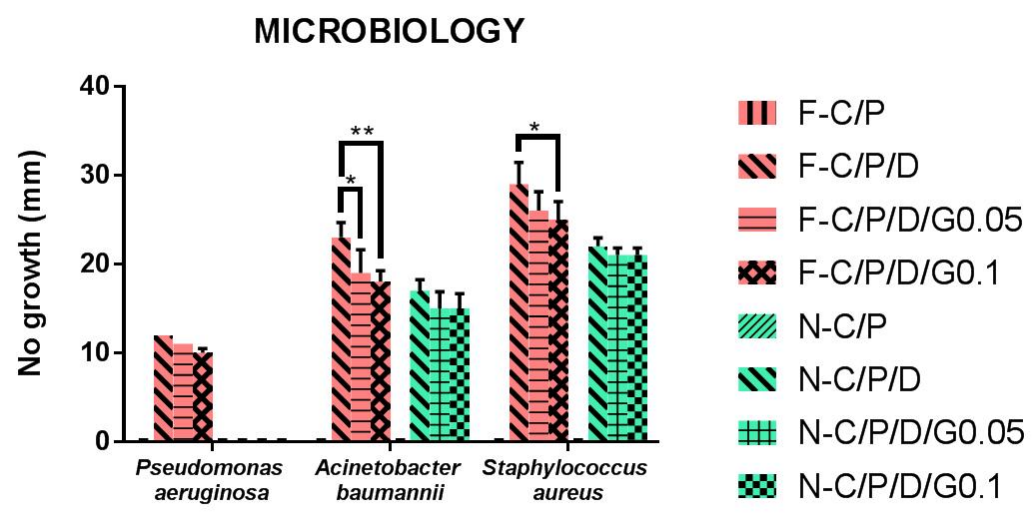
The antibacterial activity of discs from nanofiber mats and films by agar diffusion antibiogram test. n-C/P (nanofiber- Chitosan/PVA), n-C/P/D1% (nanofiber-Chitosan/PVA/Doxycycline 1%), n-C/P/D1%/G0.05% (nanofiber-Chitosan/PVA/Doxycycline 1%/ Genipin 0.05%), n-C/P/D1%/G0.1% (nanofiber-Chitosan/PVA/Doxycycline 1%/Genipin 0.1%), f-C/P (film-Chitosan/PVA), f-C/P/D1% (film- Chitosan/PVA/Doxycycline 1%), f-C/P/D1%/G0.05% (film-Chitosan/PVA/Doxycycline 1%/Genipin 0.05%), f-C/P/D1%/G0.1% (film- Chitosan/PVA/Doxycycline 1%/Genipin 0.1%).

*Water uptake*

Figure 4 shows the decrease in water uptake by film samples after the addition of doxycycline and genipin. The results showed that an increase in genipin concentration led to a significant decrease in water uptake by films (f-C/P/D_1%_/G_0.05%_ [*p *˂ 0.01], f-C/P/D_1%_/G_0.1%_ [*p *˂ 0.0001]), and nanofibers (n-C/P/D_1%_/G_0.1% _[*p* ˂ 0.05]). The addition of a cross-linking agent to the chitosan significantly reduced the water absorption and swelling properties of the polymer. Aldana *et al*. found that an increase in genipin (0.1%, 1%, and 3.25%) in chitosan/PVP films led to a reduction in swelling behaviors (21). Other studies have shown that genipin decreases the water uptake in chitosan/gelatin films (32). Another study conducted by Wang *et al.* on chitosan nanofibers showed that increasing the genipin concentration from 0.1% to 1% led to decreased water uptake (33).

*Water vapor transmission rate (WVTR)*

Control and maintenance of the moisture of the wound environment is an important factor in promoting wound healing. Figure 5 shows the results of the water vapor transmission rate for both film and nanofiber wound dressings. Doxycycline (f-C/P/D_1%_) increased the film WVTR (719 ± 15.92 g/m^2^/24 h), and genipin (f-C/P/D_1%_/G_0.1%) _decreased WVTR (598 ± 13.63 g/m^2^/24 h). The WVTRs of the nanofibers were 2797 ± 6.48 (n-C/P), 2830 ± 14.97, and 2781 ± 12.76 g/m^2^/24 h in genipin 0.05% and 0.1%, respectively. Furthermore, the addition of doxycycline (n-C/P/D_1%_) to nanofiber mats decreased the WVTR to 2728 ± 10.20 g/m^2^/24 h (*p* ˂ 0.001). A suitable wound dressing must regulate the balance of moisture between the wound surface and its environment to avoid wound exudate accumulation or wound dehydration (34). The WVTRs for the carboxyl-modified, PVA-crosslinked chitosan hydrogel film (P/C 70/30, 50/50, and 20/80) were 872, 858, and 772 g/m^2^/24 h respectively (35). Another nanofiber wound dressing with PLA had a WVTR of 3000 g/m^2^/24 h (36).

*In-vitro doxycycline release from nanofibers and films*

The release profiles of doxycycline from nanofiber mats and film are shown in Figure 6. The results indicated that the release of doxycycline from films, nanofibers, and crosslinked and uncrosslinked polymers differed. In uncrosslinked f-C/P/D_1%_, there was a burst of doxycycline released in the early times, and the release percentage reached 75% in 48 h. The release percentage of doxycycline significantly decreased with the increase of genipin in films. The doxycycline was released significantly faster from the uncrosslinked nanofiber mats than from the crosslinked mats, and the release percentage reached 85% in 48 h. The results showed that cross-linking chitosan and PVA with genipin produced a significant reduction in the release of doxycycline compared to uncrosslinked films and nanofibers (*p* ˂ 0.05). Aldana *et al.* reported that cross-linking with genipin reduced the release of propranolol hydrochloride from the film containing chitosan and polyvinyl pyrrolidone (21). The reaction between the genipin and the amino groups on the chitosan chain led to cross-linking (21, 37). Cross-linking with genipin has been shown to increase the mechanical properties of the polymeric network (21, 38) and reduce the drug release rate (21), because water uptake is associated with the formation of hydrogen bonds between water and free amino and hydroxyl groups. The presence of transverse bonds by genipin reduced the absorption of water and the drug release rate of the samples. In the current results, more than 75% of doxycycline was released in 48 h, making it a suitable wound dressing to be applied every 2 days.

*Antimicrobial susceptibility study*

The most virulent and complicated bacteria that infect wounds are *Pseudomonas aeruginosa*, *Staphylococcus aureus,* and *Acinetobacter baumannii* (39, 40). The antimicrobial activity of film and nanofiber discs showed that *Staphylococcus aureus* was the most sensitive bacterium and *Pseudomonas aeruginosa* was the most resistance bacterium. The current results also showed that film and nanofiber samples of C/P had no anti-bacterial effects. The current findings also revealed that crosslinking with genipin reduced the antibacterial effects of films and nanofibers (C/P/D/G) compared to uncrosslinked ones (C/P/D). This may be explained by the decrease in doxycycline release. Film and nanofiber samples of C/P/D_1%_ showed the greatest antimicrobial effect. Nanofibers had no antibacterial effect on *Pseudomonas aeruginosa* bacteria. F-C/P/D_1%_ showed more antibacterial effect on *Pseudomonas aeruginosa, Staphylococcus aureus,* and *Acinetobacter baumannii* compared to n-C/P/D_1%_ (*p* ˂ 0.0001). The findings of the present study were in agreement with those of Hafsa *et al.* who also showed that pure chitosan film in agar disc diffusion test produced no inhibition zone on *E*. *coli*, *S*. *aureus*, *P. aeruginosa,* and *Candida spp *(40). Another study reported that chitosan/sericin/PVA nanofibers had no antimicrobial effects against *E.coli* in an agar disc diffusion test (Figure 7) (41). This action may be due to the inability of chitosan to diffuse into agar media (42, 43). In the current study, (f-C/P/D_1%_, f-C/P/D_1%_/G_0.05%_, and f-C/P/D_1%_/G_0.1%_) could inhibit the pigment produced by *Pseudomonas aeruginosa*. These pigments, called pyocyanin and pyoverdin, are important virulence factors in the pathogenicity of *Pseudomonas aeruginosa* (44, 45). Phaechamud *et al.* demonstrated that* Staphylococcus aureus* was the most sensitive bacterium and *Pseudomonas aeruginosa* was the most resistant bacterium to doxycycline-chitosan sponge forms (17). 

The results of the current study showed the antimicrobial activity of films was more potent than that of the nanofiber mats. This may be explained by the increased rate of drug release to the agar medium and the consequent increase in antibacterial activity. The mechanism of chitosan’s antimicrobial activity is unclear, but it has been suggested that the interaction between the positive charge of chitosan and the negative charge of the bacterial cell membrane leads to destabilization of the cell membrane, permeation of intracellular components, and eventually cell death (11, 46). The physical form and morphology of chitosan affect the antimicrobial activity. Solid chitosan forms are in contact with the environment via their solid surface (11).

## Conclusion

Many factors affect the properties of nanofibers produced by electrospinning methods, including viscosity, Mw, conductivity, voltage, type of polymer, surface tension, feed rate, and concentration (23, 47-49). In the present study, electrospinning and casting methods were used to prepare films and nanofibrous mat of C/P/D and crosslink them with genipin in different concentrations. The results indicated that crosslinking with genipin decreased the diameter of nanofiber mats, water uptake, and the WVTR of films and increased the WVTR of nanofiber mats. Furthermore, the antimicrobial activities of films were more potent than those of nanofiber mats, and crosslinking with genipin decreased doxycycline release and antimicrobial activity. Genipin creates transverse bonds and reduces both the water absorption and the drug release rate from the wound dressing. Based on these results, f-C/P/D_1%_ is recommended for use as a suitable wound dressing.
